# Selective or Routine Histology of Cholecystectomy Specimens for Diagnosing Incidental Carcinoma of Gallbladder and Correlation with Careful Intraoperative Macroscopic Examination? A Systematic Review

**DOI:** 10.31557/APJCP.2021.22.3.651

**Published:** 2021-03

**Authors:** Shujaat Khan, Mohammad Azhar Rashikh, Khalil Ur Rehman, Hinanna Berjis

**Affiliations:** 1 *Department of Pathology, College of Medicine, Dawadmi, Shaqra University, Saudi Arabia. *; 2 *Department of Pharmacology, College of Medicine, Dawadmi, Shaqra University, Saudi Arabia. *; 3 *Department of Internal Medicine, College of Medicine, Dawadmi, Shaqra University, Saudi Arabia. *; 4 *Department of Anesthesiology, Ministry of Health, Dammam, Saudi Arabia. *

**Keywords:** Cholecystectomy, histology, gallbladder cancer, incidental

## Abstract

**Background:**

Selective or Routine histology of cholecystectomy specimens for benign gallbladder disease has always been a matter of debate because of the low prevalence and bad prognosis associated with gall bladder carcinoma. The objective of this study is to ascertain whether selective histology can be preferred over Routine histology without any harm.

**Methods:**

This systematic review is conducted according to PRISMA’s checklist; relevant articles were searched in the database until September 1 2020 in PubMed, Scopus, Science Direct, and Web of Science databases, manually, with search queries and without date restrictions. Studies included in this systematic review involved patients who underwent cholecystectomy for benign gallbladder disease and were diagnosed with gallbladder carcinoma incidentally either after selective or routine histology of the gallbladder.

**Results:**

A total of 24 routine or selective histology recommending studies were selected for the systematic review after following the inclusion and exclusion criteria. These studies comprised 77,213 numbers of patients and 486 numbers of Malignancies. These studies correlate the number of IGBC diagnosed histologically with the number of IGBC’s that were suspected by the surgeons intraoperative by macroscopy. Routine recommending studies show a significant number of IGBC diagnosed histologically as missed by surgeons whereas the selective recommending studies show most of the histologically diagnosed IGBC already suspected by the surgeons intraoperative. When comparing the macroscopic details of the IGBC’s between routine and selective studies, we found that there was significant overlap. Most of the findings missed by the surgeons as suspicious in routine studies were suspected by the surgeons involved in selective histology recommending studies. Thereby, favouring selective histology and emphasizing the need for careful intraoperative macroscopy for suspecting IGBC.

**Conclusion:**

Selective Histological examination of cholecystectomy specimens can be preferred if a careful intraoperative macroscopic examination is done and patient risk factors are taken into consideration.

## Introduction

One of the most frequent surgeries done worldwide is cholecystectomy (Behari and Kapoor, 2010). Cholelithiasis, an essential cause for cholecystectomy affects around 10% to 15% of the adult population living in developed countries and is the most common risk factor for gall bladder carcinoma (GBC) even though it is a benign entity (Stinton and Shaffer, 2012). Ranking fifth among the list of gastrointestinal cancers, GBC is the most common cancer of the biliary tract (Solan and Jackson, 1971). GBC is a disease with a poor prognosis (Antonakis et al., 2003). Different ethnic groups and geographical regions show the different incidence of GBC. Northern India, Pakistan, East Asia, and South America show a high rate of GBC (Behari and Kapoor, 2010; Stinton and Shaffer, 2012).

GBC has signs and symptoms similar to benign diseases of the gallbladder that are nonspecific and distinguishing it from benign disease is not possible at times (WHO Classification of Tumors Editorial Board, 2019). The most frequent radiological finding of GBC is wall thickening, which can also occur in a benign disease like Cholelithiasis (Levy et al., 2002).

Incidental gallbladder carcinoma (IGBC) is defined as a GBC diagnosed after cholecystectomy that is done for benign gallbladder disease. The term incidental was initially coined for gallbladder malignancy diagnosed as a histological surprise. The other terms used are missed or in apparent GBC (Rathanaswamy et al., 2012). Many of these patients do not show any suspicious findings intraoperative or on radiology (Waghmare and Kamat, 2014; Mittal et al., 2010).

Around 0.2% to 2.9% of all cholecystectomies done for Cholelithiasis, shows IGBC (Rathanaswamy et al, 2012; Lundgren et al., 2018). IGBC patients with stage Tis and T1a can be treated with simple cholecystectomy only, whereas stage T1b and higher require further surgical treatment (Rathanaswamy et al, 2012; Deng et al., 2015). IGBC risk factors include old age, Cholelithiasis, female sex, and obesity (WHO, 2019).

 Routine or selective histopathology of cholecystectomy specimens for detecting IGBC is a matter widely debated in countries with a varying incidence of GBM (Jayasundara and de Silva, 2013). The aggressive nature of the tumour and potential benefits by early detection emphasizes the need for routine histopathology. In contrast, the high volume of cholecystectomies and relatively low incidence of IGBC demands a selective approach which however should be so good that the risk of missing a GBC should be virtually negligible. This systematic review aims to ascertain whether selective histopathology of gallbladders could be preferred over routine histopathology for patients operated for benign gallbladder diseases to diagnose IGBM without compromising patient safety.

## Materials and Methods

This systematic review was conducted according to the Checklist of Preferred Reporting Items for Systematic Reviews and Meta-Analyses (PRISMA) guidelines.

*Information sources and search strategy*

The studies to be considered for inclusion were identified using a search strategy in each of the following electronic databases: PubMed, Scopus, Science Direct and Web of Science until September 1 2020. Keywords and terms which were used include incidental carcinoma of gallbladder, gallbladder cancer, cholecystectomy, routine histology, selective histology. A partial search for grey literature was performed using Google Scholar and by using the related articles function. The grey database and bibliographic search included all articles published without time restriction. Duplicate references were removed using the reference manager software (EndNote®, Thomson Reuters). Besides, the reference lists of selected articles were manually selected for possible relevant studies that might have been missed during the electronic database search.

*Inclusion and exclusion criteria*

Articles in English were selected with the year of publication within the last 20 years and the inclusion criteria adopted in this review were as follows: Studies in humans who underwent cholecystectomy for benign gallbladder diseases and diagnosed with Incidental carcinoma of the gallbladder on histopathology. The exclusion criteria were as follows: case reports, animal studies, literature reviews, and studies in which the diagnosis of gallbladder cancer was made preoperatively. Studies with insufficient and irrelevant data were also excluded.

*Study selection*

The selection process involved two steps. In the first step, (MAR and HB) independently checked the titles and abstracts of all the identified studies. The third reviewer, (KR) was involved in making the final decision. Studies that were not fulfilling the inclusion criteria were rejected. In the second step, the same selection process was again repeated by reviewers but after reading the whole paper. A final decision was made by (SK), after discussion with all the reviewers in case of any disagreement. 

*Data collection process*

The collection process involved a collection of the important material from each article by (HB), which was cross-checked and confirmed by (KR). Any discrepancies were solved by consensus. The data collected in these studies included the author’s name, study recommendation, study country, study year, number of cholecystectomies done, number of GBM, number of gallbladders found with suspicious intraoperative findings for GBC, number of IGBC detected as a histological surprise. Data related to the tumour stage and intraoperative/gross morphological features of tumours were also collected. Data related to patient age, sex, ethnicity, and other associated risk factors for GBC were also taken into consideration in the present study.

*Quality assessment of studies*

The quality of the studies was assessed by two authors (MAR and HB) using the Newcastle Ottawa scale which rates studies according to selection, comparability and outcome assessment with a score range from 0 to 9. (Wells GA et al., 2020).

The primary outcome was the histological diagnosis of ICGB following cholecystectomy for benign diseases of the gallbladder. Another outcome is the macroscopic finding that was associated with the histologically diagnosed IGBC.

## Results

*Study selection*

**Figure 1:**
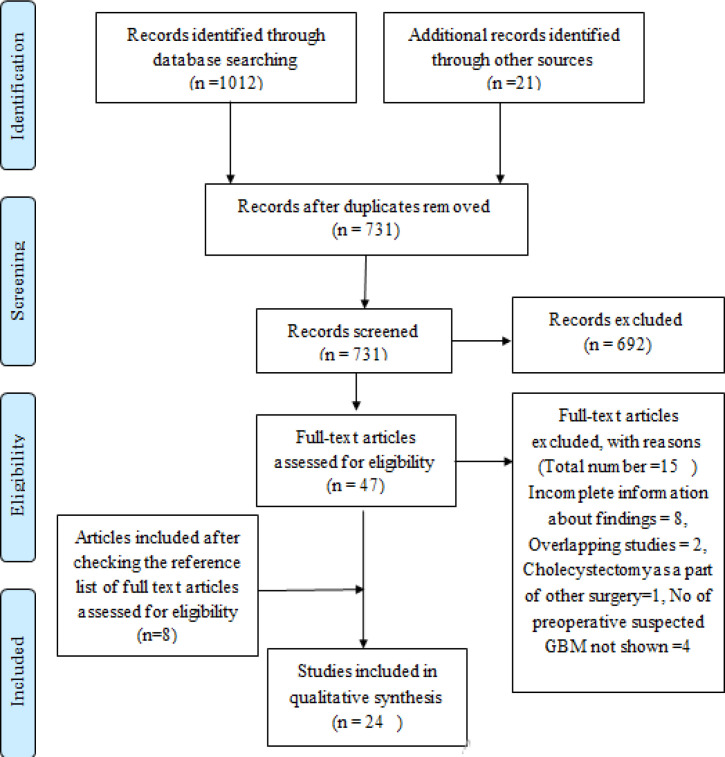
PRISMA Flow Chart for the Review

After identifying the studies through the database and other sources and removing the duplicate articles, a total of 731 studies were selected for the screening process. Of these 731 studies, 692 were excluded after screening the title and the abstract of the studies in the first step of study selection. In the second step of data selection, 15 studies were removed after the full text of the remaining 47 studies was read thoroughly. 8 studies were also added in this step by checking the references of the studies. At the end of this selection process, the total number of studies left was 24. A flowchart of the study selection process is shown in figure 1. The twenty-four studies selected by us for the review after exclusion criteria comprised 77213 numbers of patients and 486 malignancies. 

*Study characterization*

All of the studies included in the review are in the English language and conducted in countries with a different incidence of GBC. These studies have been conducted in Asia and Europe except for one study in Mexico (Romero et al., 2012) and the other in Libya (Benkhadoura et al., 2019). These studies mainly focus on comparing the intraoperative macroscopic findings with the histologically diagnosed malignancy of the cholecystectomy specimens. Based on their findings these studies either recommend selective or routine histological evaluation of cholecystectomy specimens. The summary of the study characteristics is given in Table 1.

*Risk of bias in individual studies*

To assess the study quality, two authors (MAR and HB) used the Newcastle Ottawa scale. In case of any difference, another researcher (KR) also evaluated the study quality. The Newcastle Ottawa scale rates studies according to the selection, comparability and outcome assessment with a score range from 0 to 9. All the studies included in the review were of good quality and studies with a high risk of bias were not included in the review.

*Diagnosis of Gallbladder cancer by selective histology*

After summarizing the studies, from Table 1, it is evident that the studies recommending selective histology following cholecystectomy do not identify IGBC as a histological surprise. The GBC confirmed on histology had already been suspected by the surgeon intraoperative. For example, Benkhadoura et al., (2019) from Libya report 0/4 GBM as a histological surprise with 2/4 suspected preoperatively and another 2/4 suspected intraoperative. Talreja et al., (2016) from Pakistan report 0/11 as a histological surprise and all were suspected intraoperatively. Similarly, Emmett et al., (2015) from the U.K. report 0/12 GBM as a histological surprise as all were suspected intraoperatively. Dezoysa et al., (2010) from Srilanka report 0/4 as histological surprises with 2/4 suspected preoperative and 2/4 suspected intraoperative. Similarly, Chin et al., (2012) from Malaysia reports 0/7 GBM as histological surprises.

Two selective recommending prospective studies by Tayeb et al., (2015) in Pakistan and Romero et al., (2012) in Mexico involved examination by pathologists and surgeons. Both identified 3/3 GBM that were suspected before by surgeons intraoperative and then again by the pathologist on grossing. 

*Diagnosis of Gallbladder cancer by routine histology*

From Table 1 it is also clear that the studies recommending routine evaluation of cholecystectomy specimens have a large number of histologically diagnosed IGBC without any suspicious intraoperative findings given by the surgeon. For example from India, Jeelani et al., (2019) report 28/28 IGBC detected as a histological surprise, none of which was suspected previously. Lundgren et al., (2018) from Sweden reports 153/213 IGBC that were detected only after histological evaluation. Shrestha et al., (2010) from Nepal reports 9/20 GBM detected only after histology was done. Similarly, Samad (2005) from Pakistan report 5/16 GBM detected as a histological surprise. A study from the U.K. by Patel et al., (2016) detecting 6/6 GBM as a surprise after histology. These included 2 T3 lesions, 2T2 lesions, and 1T1b lesion. 

*Details of macroscopic findings of the diagnosed Incidental carcinoma of the gallbladder*

As is clear from Table 2, many findings that were noticed by the surgeons involved in selective histology recommending studies are similar to the macroscopic findings that were not noticed by the surgeons involved in routine histology recommending studies. These were picked up on further grossing of cholecystectomy specimens before histology.

For example, Jeelani et al., (2019) from India reports 15/28 thick-walled gallbladders and 3/28 ulcerations. Lundgren et al., (2018) in Sweden noticed 60/213 gallbladders with suspicious mass, 11/213 perforated gallbladders with other findings and just 13/213 gallbladders with normal macroscopic findings. Patel et al., (2016) from the U.K. reports 4/6 gallbladders with abnormal macroscopic findings of fistula, abscess, thick-wall, disintegrated wall, and multiple calculi. Similarly Kalita et al., (2013) in India reports18/18 IGBC associated with diffuse wall thickenings, focal growth, and nodule. Jha et al., (2018) report 13/20 IGBC with abnormal gross findings like a thickened wall, ulceration. Shrestha et al., (2010) report 9/20 IGBC that on grossing had findings like fungating mass, granular mass, granular mucosae, irregular mucosae, and fibrosis wall. The above findings are similar to findings of selective recommending studies (Benkhadoura et al., 2019; Darmas et al., 2007; Dix et al., 2003) on the bases of which gallbladder cancer was suspected by surgeons involved in these studies.

**Table 1:** Summary of the Studies Recommending Routine or Selective Histology of Gallbladders

Author	Country	Design	No. of Gall bladders	No. of GBM	GBM suspected pre-operatively	GBM suspected intra- operatively	GBM suspected without histology	GBM diagnosed as histological surprise	Recommendation
Jeelani et al., 2019.	India	Retrospective	5521	28	0/28	0/28	0/28	28/28	Routine
Jha et al., 2018.	India	Retrospective	4800	20	0/20	0/20	0/20	20/20	Routine
Lundgren et al., 2018.	Sweden	Retrospective	36010	213	0/283	60/213	60/213	153/213	Routine
Patel et al., 2016.	U.K.	Retrospective	4027	6	0/6	0/6	0/6	6/6	Routine
Sangwan et al., 2015.	India	Retrospective	530	10	0/10	0/10	0/10	10/10	Routine
Siddiqui et al., 2013.	Pakistan	Prospective	220	6	0/6	0/6	0/6	6/6	Routine
Kalita et al., 2013.	India	Prospective	4115	25	0/25	0/25	7/25	18/25	Routine
Ghimre et al., 2011.	Nepal	Retrospective	783	10	0/10	0/10	0/10	10/10	Routine
Ulhaq et al., 2011.	Pakistan	Prospective	107	5	0/5	0/5	0/5	5/5	Routine
Shrestha et al., 2010.	Nepal	Retrospective	570	20	0/20	0/20	11/20	9/20	Routine
Khan et al., 2007.	India	Retrospective	472	52	0/52	0/52	44/52	8/52	Routine
Samad et al., 2005	Pakistan	Retrospective	1396	16	3/16	3+8/16	11/16	5/16	Routine
Benkhadoura et al., 2019.	Libya	Retrospective	3423	4	2/4	2+2/4	4/4	0/4	Selective
Talreja et al., 2016.	Pakistan	Retrospective	964	11	0/11	11/11	11/11	0/11	Selective
Emmett et al., 2015.	U.K.	Retrospective	4776	12	0/12	12/12	12/12	0/12	Selective
Tayeb et al., 2015.	Pakistan	Prospective	426	3	0/3	3/3	3/3	0/3	Selective
Romero et al., 2012.	Mexico	Prospective	150	3	2/3	2+1/3	3/3	0/3	Selective
Chin et al., 2012.	Malaysia	Retrospective	1375	7	5/7	5+2/7	7/7	0/7	Selective
Sajjad et al., 2012.	Pakistan	Retrospective	326	2	2/2	2/2	2/2	0/2	Selective
Byars et al., 2012.	U.K.	Retrospective	2696	7	5/7	5+2/7	7/7	0/7	Selective
Dezoysa et al., 2010	Srilanka	Retrospective	477	4	2/4	2+2/4	4/4	0/4	Selective
Mittal et al., 2010.	India	Retrospective	1305	13	0/13	0/13	13/13	0/13	Selective
Darmas et al., 2007.	U.K.	Retrospective	1452	4	1/4	1+3/4	4/4	0/4	Selective
Dix et al., 2003.	U.K	Retrospective	1292	5	3/5	3+2/5	5/5	0/5	Selective

GBM, gallbladder malignancy

**Table 2:** Comparison of Routine and Selective Histology Recommending Studies in Terms of Macroscopic Findings for Detected IGBC

Author	Number of GBM	GBM detected as histological surprise	GBM with normal macroscopy	GBM with abnormal macroscopy	Details of macroscopic findings
Routine histology recommending studies					
Jeelani et al., 2019.	28	28/28	10	18/28	15/28 Thick GB wall, 3/28 Ulceration
Jha et al., 2018.	20	20/20	7	13/20	11/20 Thick wall, 2/20 mucosal ulceration
Lundgren et al., 2018.	213	153/213	13/213	200/213	129/213 Acute, Chronic Cholecystitis, 60/213 suspicious mass or polyp, 11/213 perforation, other findings
Patel et al., 2016.	6	6/6	2/6	4/6	1/6 fistula, 1/6 thick wall, abscess, 1/6 disintegrated wall with biliary spillage, 2/6 GB inflamed with calculi, 1/6 multiple calculi
Sangwan et al., 2015.	10	10/10	10/10	0/10	6/10 multiple, mixed stones, 4/10 single, cholesterol stones
Siddiqui et al., 2013.	6	6/6	6/6	0/6	6/6 Cholelithiasis associated.
Kalita et al., 2013.	25	18/25	0/18	18/18	8/18 diffuse thickening, 10/18 focal growth, nodule
Ghimre et al., 2011.	10	10/10	7/10	3/10	1/10 thick wall, 2/10 polypoidal mass
Ulhaq et al., 2011.	5	5/5	/5	/5	5/5 associated with Cholelithiasis
Shrestha et al., 2010.	20	9/20	0/20	20/20	2/9 fungating mass, 3/9 solid grey-white mass, 1/9 granular mucosa, 1/9 irregular mucosa, 1/9 thick fibrosed wall, 1/9 contracted GB.
Khan et al., 2007.	52	8/52	NA	NA	8/8 associated with mixed stones from 1 to 4 cm
Samad et al., 2005.	16	5/16	5/16	11/16	1/16 polypoidal mass, 9/16 GB mass palpable, 5/16 enlarged lymph nodes at portahepatis
Selective histology recommending studies					
Benkhadoura et al., 2019.	4	0/4	0/4	4/4	1/4 thick wall GB, 3/4 growth, mass, 4/4 severe inflammation, adhesion
Talreja et al., 2016.	11	0/11	0/11	11/11	3/11 mucosal ulcer, 9/11 thick GB wall, 4/11 polypoidal projection
Emmett et al., 2015.	12	0/12	0/12	12/12	6/12 GB wall thick, 2/12 mass, 4/12 perforation, 1/12 fistula, 2/12 necrosis
Tayeb et al., 2015.	3	0/3	0/3	3/3	1/3 generalized wall thickness, 1/3 1cm polyp, 1/3 1.5cm growth at the fundus
Romero et al., 2012.	3	0/3	0/3	3/3	1/3 GB wall and liver induration, 1/3 induration Hartman’s pouch, 1/3 GB wall thick with visible liver metastasis
Chin et al., 2012	7	0/7	0/7	7/7	7/7 thick GB wall, 3/7 necrotic growth, 2/7 papillary projections on mucosae.
Sajjad et al., 2012.	2	0/2	0/2	2/2	1/2 diffuse thickening of GB wall, 1/2 nodular mass at the fundus.
Byars et al., 2012	7	0/7	0/7	7/7	NR
Dezoysa et al., 2010.	4	0/4	0/4	4/4	2/4 thick wall and GB mass, 1/4 adhesions, GB removed piecemeal, 1/4 gross tumour
Mittal et al., 2010.	13	0/13	0/13	13/13	NR
Darmas et al., 2007.	4	0/4	0/4	4/4	4/4 GB wall thickening, 1/4 wall necrosis, 2/4 GB mass, 1/4 empyema.
Dix et al., 2003.	5	0/5	0/5	5/5	NR

GBM, Gall Bladder Malignancy; GB, Gall Bladder; NR, Not reported

## Discussion

The diagnosis of GBC in the early stages is very important because the disease has a dismal prognosis and from the treatment point of view, curative treatment is possible only in the early stages of the disease (Agarwal et al., 2012). However, the disease has signs and symptoms that overlap with benign gallbladder diseases especially when the disease is not advanced. Most of the early GBC are thus detected in patients operated for benign gallbladder diseases and as a suspicious intraoperative finding or as a histological surprise and hence named as incidental carcinoma of the gallbladder (IGBC). Due to the huge volume of cholecystectomies done worldwide for benign gallbladder diseases and a large sum of resources spent on doing histopathology of these specimens along with the relatively low incidence of GBC, many studies suggest selective histology of cholecystectomy specimens for benign gallbladder diseases. In contrast to this, due to the aggressive nature of the tumour and potential benefits by early detection, many studies emphasize the need for routine histopathology. However, the main point of debate between the studies recommending selective and routine histology for cholecystectomy specimens is based on the identification of IGBC (Jayasundra JA and de Silva, 2013). Whereas the routine histology recommending studies state that many IGBC’s are detected by histology only and are not suspected by surgeons intraoperative, the selective histology recommending studies state that most IGBC can be suspected intraoperatively before doing histology. 

*Diagnosis of gallbladder cancer by routine evaluation*

The studies that recommend routine histology following cholecystectomy state that a large number of IGBC detected are missed by the surgeons as suspicious gallbladders and are later diagnosed as a histological surprise (Jeelani et al., 2019; Jha et al., 2018; Lundgren et al., 2018; Patel et al., 2016; Sangwan et al., 2015; V et al., 2013; Ghimire et al., 2011; Haq et al., 2011; Shrestha et al., 2010; Khan et al., 2007; Samad, 2005). Routine recommending studies highlight the grave consequences of missing many GBC by not doing routine histological evaluation especially in areas with a high incidence of gall bladder cancer and also because of the bad prognosis associated with the disease (Jeelani et al., 2019; Jha et al., 2018; Patel et al., 2016; Siddiqui et al., 2013). A study by Agarwal et al.,2012 on patients diagnosed with GBC after cholecystectomy showed that the patients who were diagnosed early because of histological evaluation of their cholecystectomy specimens showed better prognosis as compared to patients who did not have histopathology report of cholecystectomy and thus came late at an advanced stage. These studies are therefore indirectly highlighting the importance of careful intraoperative examination by surgeons and considering the incidence of GBC for deciding histological evaluation of specimens.

*Diagnosis of the gallbladder by selective evaluation*

Selective histology recommending studies state that it is unlikely to miss an IGBC as almost all of them have some suspicious macroscopic finding that could be noticed intraoperative (Benkhadoura et al., 2019; Darmas et al., 2007; Dix et al., 2003). Selective studies raise the point of wastage of money, time and resources by doing histopathology for all cholecystectomy specimens in areas and ethnic groups with a low incidence of GBC (Tayeb et al., 2015; Romero-González et al., 2012; De Zoysa et al., 2010; Mittal et al., 2010; Dix et al., 2003). These studies further state that even if a gallbladder with malignancy is looking normal or showing subtle suspicious findings intraoperative, the stage of the disease is so early like Tis and T1a, that it does not demand any treatment beyond simple cholecystectomy. This point has been proved by several studies like (Wakai et al., 2001; de Aretxabala et al., 2009; Rathanaswamy et al., 2012; Agarwal et al., 2012). Therefore these studies directly highlight the importance of careful intraoperative examination for detecting IGBC and the importance of incidence of GBC in the area while deciding histological evaluation of GB specimens.

*Details of macroscopic findings of the diagnosed Incidental carcinoma of the gallbladder for routine and selective histology recommending studies*

To the best of the author’s knowledge, this is the only systematic review done where the macroscopic findings of the gallbladders with IGBC have been compared between routine histology recommending studies and selective histology recommending studies.

As is evident from Table 2 and described under the results section, many of the macroscopic findings of GBC that were not noticed and suspected by the surgeons involved in routine histology recommending studies are noticed and suspected by the surgeons involved in selective histology recommending studies. These findings were picked up on further grossing of cholecystectomy specimens before histology.

This difference in noticing and suspecting of the gross macroscopic findings by the surgeons as malignancy emphasizes the importance of proper intraoperative examination of the specimens. If these findings would have been suspected by the surgeons involved in routine recommending studies, there would have been a significant decrease in the number of IGBC found as a histological surprise and a significant increase in the number of IGBC suspected intraoperative thus favouring selective histology of gallbladder specimens.

To summarize, in this review most of the studies that recommend routine evaluation of cholecystectomy specimens are retrospective studies done in economically poor developing countries with a relatively low doctor to patient ratio and with a high incidence of gallbladder carcinoma (Behari and Kapoor, 2010). Some of these studies report many tumours beyond stage Tis and Ta that were detected as a histological surprise and not suspected by the surgeon (Jeelani et al., 2019; Jha et al., 2018; Patel et al., 2016; Siddiqui et al., 2013). However, early-stage carcinoma Tis, T1a do not require any additional treatment then cholecystectomy and late stages like T3 shows macroscopic findings as the gallbladder wall has been breached, but for many T2/T3 tumours showing subtle findings or suspicious lesions being masked by benign pathology, need for careful intraoperative macroscopic examination arises.

Cholecystectomy being one of the commonest surgeries performed, it is likely that some suspicious findings are missed by surgeons doing a large number of cholecystectomies in the absence of any well-defined guidelines for gallbladder examination. Such findings are later noticed while grossing of the gallbladder specimens before histology. This may be the cause of more IGBC detected as a histological surprise rather than intraoperative suspected. In such a case, guidance by the associated pathologist will certainly improve the quality of intraoperative examination of gallbladders for ruling out IGBC. With this protocol, a substantial chance of missing intraoperative IGBC will be reduced and the negligible risk of missing a few IGBC could be weighed against the huge benefits in terms of time, money and resources saved from being wasted on near-normal gallbladders. These human and material resources could then be used in other areas where they are needed more. This is especially true in the case of developing and underdeveloped countries with limited health resources and low-income charitable hospitals. This approach could be implemented and will be useful to surgeons as well who find it difficult to decide about gallbladders to be compulsorily sent to the overburdened histopathology labs due to various precluding factors.

 In our opinion because of the huge volume of cholecystectomies being performed worldwide, a selective approach should be taken into consideration. This is especially true for countries with a low incidence of GBC and maybe for those economically poor countries where the incidence of GBC is high and implementing routine histology for every cholecystectomy is not practical. A study in one such country India by Agarwal et al., (2012) in a centre that receives cases of gallbladder cancer is evidence that a huge number of gallbladder carcinoma come at a later stage because histopathology being not done by the surgeons for every cholecystectomy probably due to various reasons like unavailability of histopathology services, cost, unawareness regarding IGBC prognosis and so on. This is despite the directions of doing histopathology for every case of cholecystectomy (Royal College of Pathologists, London, 2005). 

Thus as per this systematic review, in areas where the incidence of GBC is low, selective evaluation of gallbladders with benign diseases should be preferred. This supports the previously done review by (Jamal K et al., 2014). The findings of our systematic review further strengthen the view of another review article by (Jayasundra et al., 2013) that selective evaluation may be considered in areas with a low incidence of GBC. 

In addition to this, the current review also showed the importance of careful intraoperative macroscopy for suspecting IGBC intraoperative. This further strengthens prerequisite criteria of normal macroscopy of gall bladder for selective histology by previous review (Jamal et al., 2014). Careful Intraoperative macroscopy significantly decreases the number of IGBC detected as a histological surprise and thus favours selective histology of cholecystectomy specimens, especially in low incidence areas. For areas with a high incidence of GBC and where most of the routine recommending studies have been done, a selective approach might be implemented if careful intraoperative macroscopy is done.

We hope that soon, many large prospective studies will be done involving surgical and pathological experts in areas where the incidence of GBC is high, to assure the safety of selective histology after careful intraoperative examination of gallbladders.

*Limitations*

Our study is limited to PubMed, Scopus, Science Direct and Web of Science database searches with articles in the English language. So, all the studies conducted in the field and those published in non-indexed local journals and articles in a language other than English might not be covered by us. Besides most of the studies included in the review are retrospective studies and only some of the studies are prospective studies.

In conclusion, to conclude based on this review we feel that for countries with a low incidence of GBC, selective histopathology of gallbladders should be preferred after careful macroscopic examination of the gallbladder is done and patient risk factors are taken into consideration. For those economically poor countries with a high incidence of GBC, selective histopathology is a practical decision provided a careful macroscopic examination of the gallbladder is done and the GBC risk factors are taken into consideration. 

## Author Contribution Statement

The authors confirm contribution to the paper as follows: study conception and design: Shujaat Khan and Mohammad Azhar Rashikh; data collection: Hinanna berjis and Khalil ur Rehman; analysis and interpretation of results: Shujaat Khan, Mohammad Azhar Rashikh and Khalil ur Rehman; draft manuscript preparation: Shujaat Khan and Hinanna Berjis. All authors reviewed the results and approved the final version of manuscript.
